# The economic burden of advanced gastric cancer in Taiwan

**DOI:** 10.1186/s12913-017-2609-1

**Published:** 2017-09-16

**Authors:** Jihyung Hong, Yiling Tsai, Diego Novick, Frank Chi-huang Hsiao, Rebecca Cheng, Jen-Shi Chen

**Affiliations:** 1 Department of Healthcare Management, College of Social Science, Gachon University, Seongnam, South Korea; 2 Eli Lilly and Company, Taipei, Taiwan; 3 Eli Lilly and Company, Windlesham, Surrey, UK; 4 Linkou Chang Gung Memorial Hospital and Chang Gung University, Tao-Yuan, Taiwan

**Keywords:** Gastric cancer, Burden of illness, Cost, Taiwan

## Abstract

**Background:**

Gastric cancer is one of the leading causes of cancer-related deaths in both sexes worldwide, especially in Eastern Asia. This study aimed to estimate the economic burden of advanced gastric cancer (AGC) in Taiwan.

**Methods:**

The costs of AGC in 2013 were estimated using resource use data from a chart review study (*n* = 122 with AGC) and national statistics. Annual per-patient costs, where patients’ follow-up periods were adjusted for, were estimated with 82 patients who had complete resource use data. The costs were composed of direct medical costs, direct non-medical costs (healthcare travel and caregiver costs), morbidity costs, and mortality costs. Relevant unit costs were retrieved mainly from literature and national statistics, and applied to the resource use data. A broad definition of morbidity and mortality costs was employed to value the productivity loss in patients with unpaid employment, economically inactive and unemployed as well as the life years after the age of retirement. Their narrow definitions were also used in sensitivity analyses, using age- and/or sex-specific employment rates. Forgone future earnings/productivity loss were discounted at 3%. Annual per-patient costs were projected to estimate the total costs of AGC at the national level with an estimated number of patients with AGC (*N* = 2611) in Taiwan in 2013.

**Results:**

The mean age of the 82 patients was 59.3 (SD: 11.9) years, and 67.1% were male. Per-patient costs were US$26,431 for direct medical costs, US$4669 for direct non-medical costs, US$5758 for morbidity costs, and US$145,990 for mortality costs (per death). These per-patient costs were projected to incur total AGC costs of US$423 million at the national-level. Mortality costs accounted for 77.3% of the total costs, followed by direct medical costs (16.3%), morbidity costs (3.6%), and direct non-medical costs (2.9%).

**Conclusion:**

AGC was found to exert a significant economic burden in Taiwan, incurring US$423 million in 2013. This represents about 0.08% of the Taiwanese economy. Mortality costs appeared to be the single greatest contributor to the burden, followed by direct medical costs. Early detection and providing effective treatments will help to reduce its burden on patients, caregivers and society as a whole.

A poster of this study was presented at the 2016 American Society of Clinical Oncology (ASCO) Gastrointestinal Cancers Symposium in San Francisco, CA, USA.

## Background

Despite a substantial decline in both incidence and mortality over the past decades, gastric cancer (GC) still remains an important public health burden worldwide [1]. It is the fifth most common cancer with more than one million new cases every year [2]. The incidence of GC is particularly high in Eastern Asia, followed by Central and Eastern Europe, and South America [2]. In Taiwan, GC ranks eighth in cancer incidence with an age-adjusted incidence rate of 11.1 (crude rate: 16.3) per 100,000 persons in 2012 [3].

GC is characterised by the high variability of non-specific symptoms such as dyspepsia, weight loss and anaemia, which makes early diagnosis difficult. Once GC is detected, it is typically at an advanced stage, making treatments even more burdensome and costly. Notably, the 5-year survival rate at this stage is reported to be less than 25% [4], resulting in additional 17.9 million disability-adjusted life years (DALYs) [5]. Similarly in Taiwan, about 60% of GC patients are diagnosed at an advanced stage [6, 7], making GC rank sixth in cancer mortality with an age-adjusted mortality rate of 6.8 (crude rate: 10.2) per 100,000 persons in 2012 [3].

A limited number of studies have demonstrated a substantial economic burden of GC in both Western and non-Western countries. For instance, Haga et al. estimated the cost of GC in Japan, using national statistics [8]. They reported the cost of GC to be ¥1114.2 billion (about US$11 billion) in 2008 including both direct and indirect (i.e., productivity loss related to morbidity and premature death) costs. Another study, which was conducted in South Korea, estimated the cost of GC to be about US$2.8 billion in 2009 [9]. The cost of GC was also found to be substantial even in the United States, where the incidence of GC is relatively low [10]. Mariotto et al. [11] estimated the direct medical cost of GC care to be US$1.82 billion in 2010, but did not report the size of GC-related productivity loss (i.e., morbidity and mortality costs). To the authors’s knowledge, there is no published study that thas estimated the full economic burden of GC in Taiwan. Nevertheless, the findings from Li et al.’s study (2014) has indicated a considerable burden of GC in the country [12]. The study estimated and compared the per-patient direct medical costs of initial cancer care by type of cancer, using the National Health Insurance data [12]. Among five cancers (GC, lung cancer, liver cancer, colorectal cancer, breast cancer) analysed, GC and lung cancer exhibited the greatest increase in the per-patient direct medical costs between 1996 and 2007. Their costs also appeared to be the highest with US$10,780 (for GC) and US$10,681 (for lung cancer) in 2007, respectively.

This study therefore aimed to quantify the economic burden of GC at both patient-level and national-level in Taiwan; this will help better prioritise and allocate resources for cancer management. Given that GC is often detected at an advanced stage and treatment varies with stage, this study focused on the burden associated with advanced gastric cancer (AGC), using data from a chart review study and national statistics.

## Methods

### Patients and data sources

The present study estimated the economic burden of AGC in Taiwan. There is, however, no single definition of AGC uniformly used in the literature [13]. This study therefore defined AGC, consistent with that used in the main data source [14]. That is, AGC was defined as metastatic and/or locally recurrent, unresectable gastric cancer, including cancer of the stomach and gastroesophageal junction (GEJ) with adenocarcinoma histology.

The main data source for this COI study was a retrospective chart review study conducted in Taiwan, which provided data on treatment patterns and resource utilisation among 122 patients with AGC [14]. Physicians were randomly selected from a panel of oncologists, and participated in the study upon their agreement (*n* = 37). Each participating physician was asked to provide de-identified patient-level data from medical charts for up to 10 randomly selected patients diagnosed with metastatic and/or locally recurrent, unresectable gastric cancer (i.e., AGC), including cancer of the stomach and gastroesophageal junction (GEJ) with adenocarcinoma histology. Data were collected from 1 April 2013 to 8 July 2013 using a secure online chart abstraction instrument.

Patients were eligible for inclusion in this chart review study if they (1) had a diagnosis of AGC, including GEJ with adenocarcinoma histology, on or after 1 January 2009; (2) had received first-line treatment with platinum/fluoropyrimidine after AGC diagnosis, were eligible for second-line therapy, and initiated either second-line therapy or best supportive care (BSC) after the first-line treatment; (3) were aged at least 18 years old at the diagnosis of AGC; (4) were not participating in other clinical trials (except for patient registries or observational studies) after AGC diagnosis; and (5) did not have other primary malignant tumours.

The details of this chart review study have been published elsewhere [14].

### Cost definition and estimation

The economic burden of AGC in 2013, expressed in US$ (the market exchange rate of 1 US$ = 30 NT$ [15]), was estimated at both patient-level and national-level from a (limited) societal perspective and included (1) direct medical costs; (2) direct non-medical costs; (3) morbidity costs; and (4) mortality costs [16, 17]. The market exchange rate was used when converting New Taiwan dollars into US dollars to be consistent with previous Taiwanese costing studies [12, 18–20]. It should however be noted that the use of purchasing power parity (PPP) could be more appropriate for an international comparison because it is the rate of currency conversion that adjusts for different price levels between countries. The implied PPP conversion rate for Taiwan was 1 US$ = 14.9 NT$ in 2013 [21], and this conversion rate could therefore be considered when comparing the burden of AGC across countries.

**Fig. 1:**
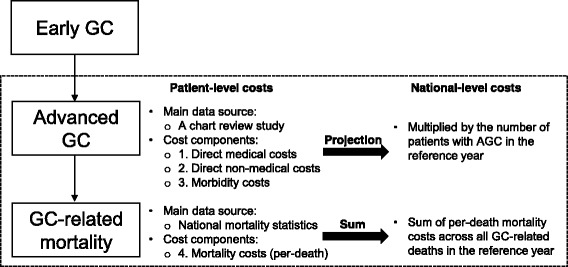
Process of data extraction and cost estimation. Abbreviations: GC, Gastric Cancer

The mean per-patient costs were first calculated and projected to estimate total costs of AGC in Taiwan based on prevalence data. The per-patient costs were calculated based mainly on the chart review data. Of the 122 patients included in the chart review study, this COI study included only those patients having a follow-up of at least 50 days (*n* = 118). Although this is an arbitrary cut-off point, this can help more accurately estimate “annual” per-patient costs, minimising the loss of eligible patient observations. Different follow-up periods were also adjusted for to estimate the annual costs. The final cost analysis was, however, based on 82 patients who had complete resource utilisation data. The per-patient costs were multiplied by the prevalence of AGC in 2013. Given the lack of prevalence data however, the number of patients with AGC in 2013 (*N* = 2611) were derived from the number of patients with GC in 2012 (*N* = 4609) and percentages of stage III (27.19%) and stage IV (29.47%) of newly diagnosed GC cases in 2012 in Taiwan [6, 7]. The process of data extraction and cost estimation was delineated in Fig. 1.

Resource utilisation and per-patient costs were summarised with descriptive statistics (e.g., mean, median and percentage) using STATA/SE 11.2. They were also summarised and compared by type of treatment (second-line treatment versus BSC) following the first-line treatment, using both parametric and non-parametric tests. Both tests provided similar results, and t-test results were reported in the result section.

#### Direct medical costs

1 $$ D{MC}_i={InC}_i+{OutC}_i+{ChemoC}_i\ \left(\mathrm{for}\ \mathrm{a}\ \mathrm{patient}\ i\right) $$

Direct medical costs (DMC_*i*_) included (1) inpatient costs (InC_*i*_) associated with AGC-related overnight inpatient stay and other inpatient visits (hospice units, skilled nursing facilities, oncology-oriented inpatient rehabilitation clinics) (e.g., for the management of disease symptoms, toxicities and comorbidities); (2) outpatient costs (OutC_*i*_) associated with AGC-related outpatient clinic visits (outpatient rehabilitation clinic visits, oncologist clinic visits, gastroenterologist clinic visits, other clinic visits, nutritional support visits, and home visits) (e.g., for the management of disease symptoms, toxicities and comorbidities); and (3) chemotherapy-related costs (ChemoC_*i*_) (.e., drug costs and related administration costs). Different unit costs were applied to each type of medical resources (see Table 1). Unit costs were inflated to the reference year (2013) if needed, using the annual change of consumer price indices [22]. The chart review study did not collect inpatient and outpatient resource utilisation data related to regularly scheduled chemotherapy infusion. These costs were therefore included as part of chemotherapy administration costs.

**Table 1:** Unit costs of major medical resources

Types of medical resources	Unit costs (US$)^a^
Overnight inpatient stay	311 [18]
Hospice unit stay	103 [18]
Skilled nursing facility stay	50 [42–44]
Oncology-oriented inpatient rehabilitation clinic stay	311 [18]
Outpatient clinic visits	155 [45]
Nutritional support visits	8.5 [46–50]
Home visits	69 [51]

^a^ Each unit cost was taken from the reference listed with some price adjustments

Chemotherapy-related costs were composed of drug costs and related administration costs. Drug costs were calculated based on type of chemotherapy and number of cycles collected in the chart review study. Given the paucity of resource utilisation data, recommended dose and administration were separately collected for each chemotherapy treatment and applied to the type of chemotherapy and the number of cycles to calculate chemotherapy drug costs. The recommended dose, which was expressed either in mg/m^2^ or mg/kg, was converted to mg, using the mean body surface area of 1.575 and the mean body weight of 56.2 kg taken from the subgroup of Taiwanese patients with advanced gastric or gastro-oesophageal junction adenocarcinoma included in a randomised controlled trial [23]. Drug administration costs were calculated based on the following activities: drug dispensing, intravenous (IV) administration, IV drip/pump, other basic medical services provided during inpatient admission, and chemotherapy-related routine examinations (complete blood count, biochemistry profile, carcinoembryonic antigen test, plain abdomen X-ray every month and CT scan every 2.5 month).

#### Direct non-medical costs

2 $$ {DNMC}_i=\left({InN}_i+{OutN}_i+{ChemoIN}_i\right)\times (UCt)+\left(1\times {IndaysN}_i+0.5\times {OutN}_i+1\times ChemoIN\_{A}_i+2\times ChemoIN\_{B}_i\right)\times (UCwage)\ \left(\mathrm{for}\ \mathrm{a}\ \mathrm{patient}\ i\right) $$

Direct non-medical costs (DNMC_*i*_) included transportation costs and caregiver costs. For transportation costs, the single round-trip costs (UCt = US$13.8 [24]) were applied to the number of inpatient (InN_*i*_) and outpatient (OutN_*i*_) visits as well as the number of chemotherapy drug administrations (injection forms only) (ChemoIN_*i*i_). For caregiver costs, it was assumed that one caregiver was needed for one patient during inpatient stays, outpatient visits, and chemotherapy drug administrations (injection forms only). One day was assumed for an inpatient day (IndaysN_*i*_), a half day was assumed for an outpatient visit (OutN_*i*_), and one (ChemoIN_A_*i*_) or two (ChemoIN_B_*i*_) days were assumed for 1 injection-form chemotherapy administration, depending on the type of chemotherapy. The average daily wage in the industry of support services (UCwage = US$48 [25]) was applied to the number of care-giving days.

#### Morbidity costs

3 $$ {MorbidityC}_i=\left(1\times {IndaysN}_i+0.5\times {OutN}_i+1\times ChemoIN\_{A}_i+2\times ChemoIN\_{B}_i\right)\times \left({UCwage}_j\right)\times \left({E}_j\right)\ \left( for\ a\  patient\ i\right)\left(j=1\ \mathrm{or}\ 2,\mathrm{gender}\right) $$

There are potentially three types of productivity losses associated with morbidity, which are (i) productivity loss due to cancer treatment, (ii) productivity loss due to being sick (absenteeism and presenteeism), and (iii) job losses due to cancer treatment and/or being sick. Given the lack of information, the present analysis focused only on productivity loss due to cancer treatment (i.e., time costs). It was defined as the value of lost productivity due to patients’ time spent on cancer treatment (MorbidityC_*i*_). The number of lost work days due to cancer treatment was calculated by summing the number of inpatient days (IndaysN_*i*_), the number of outpatient visits (OutN_*i*_) multiplied by 0.5 (i.e., half-day), and the number of injection-form chemotherapy administrations multiplied by 1 (ChemoIN_A_i_) or 2 (ChemoIN_B_*i*_) days. Given limited information, only sex-specific average daily wage was applied to all age groups (UCwage_*j*_ = US$74 for male and US$62 for female [25]).

In a sensitivity analysis, sex-specific average employment rates (E_*j*_) were further applied [25], and zero costs were applied to those patients above 69 years old (>69). The rational for this sensitivity analysis is explained in the section below.

#### Mortality costs

4 $$ {MortalityC}_i={\sum}_j{\sum}_k{\sum}_{l=1}^n\left({N}_{jk}\times \frac{Y_{jk\left(t+l\right)}\times \left({E}_{jk\left(t+l\right)}\right)}{{\left(1+r\right)}^l}\right)/{\sum}_j{\sum}_k{N}_{jk} $$

MortalityC_*i*_ = the average cost per death, N_*jk*_ = the number of sex(j)- and age(k)-specific deaths, l = 1,2,…,n(n = the number of years lost), t = age at the time of death, r = discount rate, Y_*jk(t+l)*_ = sex- and age-specific average annual income at the time of t + l, E_*jk(t+l)*_ = sex- and age-specific employment rate at the time of t + l.

Mortality costs are, in general, defined as “future income loss” due to premature death, and therefore often calculated within the working population from the time of death till the age of retirement (65 or 69 years old). However, recent studies have criticised this approach as “inaccurate” and “unethical”, and therefore valued the years even after the age of retirement [9, 26]. The present study adopted this recent approach (i.e., broad definition) and therefore placed economic value (Y_*jk(t+l)*_) on a person’s life till the end of his or her life expectancy since the time of death (*n* = the difference between the age of death and life expectancy at the time of death) [27]. A traditional approach (i.e., narrow definition) was instead taken in a sensitivity analysis, and future income losses between the time of death and the age of 69 were calculated within the expected working population, using sex- and age-specific annual income (Y_*jk(t+l)*_) and employment rates (E_*jk(t+l)*_) [28, 29]. All potential future earnings were discounted at 3% (*r*) to calculate their present value.

To be consistent, this narrow definition was also applied to morbidity costs in the sensitivity analysis as aforementioned.

## Results

### Patient characteristics

Table 2 describes the characteristics of the 118 AGC patients included in the chart review study. The mean age of the patients was 59.6 years (standard deviation [SD]: 12.9), and 63.6% were male (Table 2). These patients had the mean follow-up of 397.1 days (SD: 300.0) (median: 291.5 days). About one in five (17.8%) had a history of Helicobacter pylori infection, and only a small fraction (4.2%) had a family history of GC.

**Table 2:** Patient characteristics

Patient characteristics	Total (n = 118)
Age, mean ± SD	59.6 ± 12.9
Follow-up days, mean ± SD	397 ± 300
Being male	63.6%
Smoking history	
Non-smoker	64.4%
Current smoker	17.8%
Former smoker	12.7%
Unknown	5.1%
Alcohol consumption	
No alcohol use	67.0%
Light to moderate	25.4%
Heavy	2.5%
Unknown	5.1%
A history of Helicobacter pylori infection	
Yes	17.8%
No	43.2%
Unknown	39.0%
A family history of gastric cancer	
Yes	4.2%
No	84.8%
Unknown	11.0%
Patients alive at the time of data collection	43.2%

### Resource utilisation

Of the 118 patients, 66.1% (*n* = 78) had second-line treatment, and the rest (*n* = 40) had best supportive care following the first-line treatment. Patients with second-line treatment had a longer follow-up than those with BSC (mean [SD]: 448.5 days [320.0] vs. 296.9 days [228.6]; median: 326.5 days vs. 211.5 days).

Patients all together, on average, had 29.0 inpatient days (SD: 37.3) and 21.1 outpatient visits (SD: 15.4) during follow-up (Table 3). This resource utilisation remained largely constant when different follow-up periods were adjusted for to estimate the annual mean resource utilisation (mean [SD]: 33.4 inpatient days [44.4] and 21.3 outpatient visits [14.0]).

**Table 3:** Resource utilisation among patients with AGC during follow-up

Resource utilisation	n	Total (n = 118)	BSC (n = 40)	2nd-line TX (n = 78)
Mean follow-up days (SD)	118	397.1 (300.0) [Median: 291.5]	296.9 (228.6) [Median: 211.5]	448.5 (320.0) [Median: 326.5]
Inpatient stay (days), mean(SD)				
Overnight inpatient stay	118	19.6 (22.5)	17.9 (26.0)	20.5 (20.6)
Hospice unit stay	99	2.4 (5.1)	2.0 (3.2)	2.6 (5.9)
Skilled nursing facility stay	96	2.4 (20.4)	7.2 (36.4)	0.2 (1.1)
Oncology-oriented inpatient ^a^ rehabilitation clinic stay ^a^	96	7.5 (18.0)	12.5 (25.1)	5.2 (13.3)
Total	94	29.0 (37.3)	34.8 (52.2)	26.4 (28.4)
Outpatient visits (visits), mean(SD)				
Outpatient clinic visits	86	19.6 (14.0)	19.1 (16.9)	19.8 (13.0)
Nutritional support visits	104	1.3 (2.2)	1.4 (1.4)	1.3 (2.5)
Home visits	97	0.7 (1.8)	0.7 (1.0)	0.7 (2.1)
Total	84	21.1 (15.4)	19.9 (17.6)	21.4 (14.7)

Abbreviations: *AGC* Advanced Gastric Cancer, *BSC* Best Supportive Care, *SD* Standard Deviation, *TX* treatment

^a^Both parametric and non-parametric tests indicated no statistically significant differences in resource utilisation between patients with BSC and patients with second-line treatment following the first-line treatment, except for skilled nursing facility stay (Mann-Whitney test *p*-value = 0.019). [Also in oncology-oriented inpatient rehabilitation clinic visits at *p* < 0.1 (t-test p-value = 0.066, Mann-Whitney p-value = 0.061)

Resource utilisation was also analysed by type of treatment following the first-line treatment. Despite a longer follow-up, patients with second-line treatment had numerically fewer inpatient visits than those with BSC during follow-up (mean inpatient days [SD]: 26.4 days [28.4] vs. 34.8 days [52.2]; *p*-value = 0.314). The number of outpatient visits was very similar between the two groups (mean [SD]: 21.4 visits [14.7] for second-line treatment vs. 19.9 visits [17.6] for BSC; p-value = 0.693).

### Economic burden of AGC

Per-patient costs were estimated with 82 patients having complete resource utilisation data. The characteristics of these patients were largely similar to those of the 118 patients (mean age [SD]: 59.3 years [11.9]; male %: 67.1%), although follow-up was longer in these 82 patients (443.8 days [SD: 315.6]). Nevertheless, the level of resource utilisation was largely similar when comparing the items with all available observations and the items with complete resource utilisation data (data not shown).

**Fig. 2:**
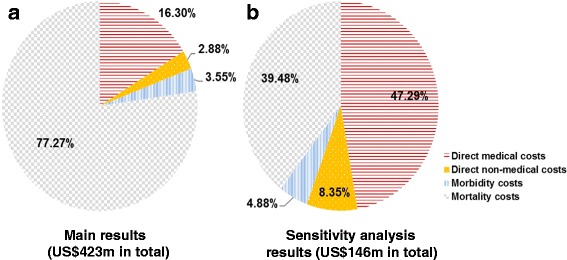
Cost breakdown for total economic burden of AGC in Taiwan. **a**. Main results (US$423 m in total) . **b**. Sensitivity analysis results (US$146 m in total). Note: morbidity and mortality costs were estimated within the potentially working population (i.e., narrow definition) in the sensitivity analysis. Abbreviations: AGC, Advanced Gastric Cancer; m, million

The total cost of AGC was estimated based on per-patient costs and prevalence of AGC in 2013. The full economic burden of AGC appeared to be substantial with US$423 million at the national-level (Table 4). Mortality costs accounted for 77.3% of the total costs, followed by direct medical costs (16.3%), morbidity costs (3.6%), and direct non-medical costs (2.9%) (see Fig. 2a). Per-patient costs were estimated to be US$145,990 (per death) for mortality costs, US$26,431 (SD: 15,322) for direct medical costs, US$5758 (SD: 3053) for morbidity costs and US$4669 (SD: 2462) for direct non-medical costs. However, the size of morbidity and mortality costs differed substantially depending on the definition employed. With a narrow definition in the sensitivity analysis where morbidity costs and mortality costs were limited to the potentially working population, direct medical costs accounted for 47.3% of the total costs (US$146 million) of AGC in 2013, followed by mortality costs (39.5%), direct non-medical costs (8.4%), and morbidity costs (4.9%) (see Fig. 2b). The bulk of the direct medical costs were from treatment with chemotherapy (59.6%) (drug costs: US$10,912 [SD: 9420]; administration/admission costs: US$3941 [SD: 3226]; routine examination: US$888 [SD: 300]), followed by inpatient stay (28.6%), and outpatient visits (11.8%).

**Table 4:** Total costs of AGC (US$) in 2013 (*n* = 82 with complete RU data)

Type of costs	Per patient (US$) ^a^	At nation-level (US$)
Direct medical costs	26,431 (15,322)	69,022,360
Inpatient costs	7570 (8671)	19,769,724
Outpatient costs	3120 (1950)	8,146,542
Chemotherapy related costs	15,741 (10,963)	41,106,095
- Drug costs	10,912 (9420)	28,496,062
- Drug administration costs	3941 (3226)	10,291,600
- Costs of routine examinations	888 (300)	2,318,457
Direct non-medical costs	4669 (2462)	12,193,870
Transportation costs	702 (372)	1,834,263
Caregiver costs	3967 (2141)	10,359,607
Morbidity costs	5758 (3053)	15,035,791
Mortality costs	145,990^b^	327,162,561
Total costs of AGC	–	423,414,583

Abbreviations: *AGC* Advanced Gastric Cancer, *RU* Resource Utilisation

^a^ Different follow-up periods were adjusted for to estimate “annual” costs. Data were expressed in mean with standard deviation if not specified

^b^ Costs per death due to gastric cancer

Table 5 shows additional per-patient costs by type of treatment following the first-line treatment. The annual per-patient costs (i.e., direct medical costs, direct non-medical costs and morbidity costs), which adjusted for patients’ follow-up periods, were numerically greater in patients with BSC than in patients with second-line treatment.

**Table 5:** Mean per-patient costs (US$) by type of treatment initiated following first-line therapy (n = 82 with complete RU data)

Type of costs	Costs during follow-up		Annual costs^a^	
	BSC (*n* = 20)	2nd-line TX (*n* = 62)	BSC (n = 20)	2nd-line TX (n = 62)
Mean follow-up days (SD)	401 (251) [Median: 305]	458 (334) [Median: 338]	–	–
Direct medical costs, mean(SD)	24,911 (11,661)	25,938 (17,279)	27,583 (15,051)	26,059 (15,512)
Inpatient costs	8386 (13,127)	7741 (8913)	8119 (11,093)	7393 (7835)
Outpatient costs	3074 (2715)	3116 (2078)	3340 (2508)	3049 (1752)
Chemotherapy related costs	13,451 (6223)	15,081 (13,174)	16,124 (10,140)	15,617 (11,291)
- Drug costs	9240 (5184)	10,570 (11,974)	11,205 (7665)	10,817 (9975)
- Drug administration costs	3355 (3017)	3524 (2520)	4045 (4224)	3907 (2874)
- Costs of routine examinations	856 (537)	987 (669)	874 (311)	892 (299)
Direct non-medical costs, mean(SD)	4232 (2227)	4476 (2655)	4815 (2813)	4622 (2362)
Transportation costs	629 (401)	678 (461)	718 (432)	698 (354)
Caregiver costs	3602 (1925)	3798 (2250)	4098 (2435)	3925 (2057)
Morbidity costs	5325 (2824)	5531 (3276)	6042 (3538)	5666 (2905)

Abbreviations: *AGC* Advanced Gastric Cancer, *BSC* Best Supportive Care, *RU* Resource Utilisation, *SD* Standard Deviation, *TX* Treatment

Note: both parametric and non-parametric tests showed no statistically significant differences in per-patient costs (in terms of both follow-up and annual costs) between patients with BSC and patients with second-line treatment following first-line treatment

^a^Different follow-up periods were adjusted for to estimate “annual” costs

## Discussion

This study estimated the economic burden of AGC in Taiwan at both patient-level and national-level in 2013. AGC was found to exert a significant economic burden in Taiwan, incurring a total of US$423 million at the national-level in 2013. This estimate represents 0.08% of the Taiwanese economy (nominal gross domestic product [GDP] in 2013: about US$512 billion [30]).

### Comparison of AGC burden with other neighbouring countries

There are very few COI studies that have estimated the economic burden of GC in other countries [8, 9], and more importantly, none of these studies have estimated the burden of AGC. It is therefore difficult to make a direct comparison among these studies. Notably, the burden of AGC in our study is likely to be smaller than that of GC at the national-level, if other things being equal, because AGC patients are a subset of GC patients. In addition, differences in study methodologies and data employed further challenge the direct comparison among COI studies. These difficulties should therefore be fully taken into account when making such comparisons.

While our estimate confirms a substantial burden of AGC in Taiwan, the burden, as a share of GDP, appears to be smaller than those reported in other neighbouring countries like Japan and South Korea where the prevalence of GC is particularly high. For instance, Haga et al. estimated the cost of GC in 1996, 2002, and 2008 in Japan, using national statistics [8]. They reported the burden of GC to be ¥1114.2 billion (about US$11 billion) in 2008, which is equivalent to about 0.23% of Japan’s nominal GDP in that year [31]. As in our study, mortality costs accounted for 72.4% of GC costs in 2008. However, despite such a high burden of GC in Japan, the study showed a downward trend of GC burden, from ¥1293.5 billion (about US$12 billion) in 1996 to ¥1114.2 billion in 2008. The decrease in mortality cost was reported to be the major contributing factor. It should be noted that the number of cancer deaths remained constant between 1996 (50,161 deaths) and 2008 (50,156 deaths), but the increased proportion of people aged 65 or older (from 70.1% in 1996 to 80.7% in 2008) reduced the human capital value and thereby mortality costs.

There is also a Korean study, which estimated the economic burden of cancer in 2009 [9]. GC was identified as a single most costly cancer with a total of US$2.8 billion (US$677 million for medical costs, US$518 million for non-medical costs, US$814 million for morbidity costs, and US$827 million for mortality costs). This is equivalent to about 0.31% of the country’s nominal GDP in 2009 [31], which is fairly similar to the burden of GC in Japan aforementioned. Nevertheless, the contribution of cost components differed considerably between the two studies. Mortality costs accounted for more than two-thirds (72.4%) of the total costs in Japan, but only about one-third (29.2%) of the total costs in South Korea. The costing method used in our main analysis is similar to that employed in this Korean study [9]; both studies have placed an economic value until patient’s life expectancy for mortality costs. However, mortality costs in our study appeared to take up over two-thirds of the total costs, despite a lower incidence of GC in Taiwan. This reaffirms that premature death due to AGC places a significant burden on patients, caregivers and society as a whole in Taiwan.

### Economic burden of AGC: Key cost drivers

As aforementioned, mortality costs were found to be the single greatest contributor to the AGC burden in Taiwan. The costs accounted for more than two-third of the total costs (77%), followed by direct medical costs (16%), morbidity costs (4%), and direct non-medical costs (3%). These findings indicate that improving survival of GC patients can substantially reduce the economic burden of AGC in Taiwan. Notably, Taiwan has a mortality-to-incidence ratio of about 0.59–0.63 [32–34]. This is slightly smaller than those observed in many other countries, but greater than the ratios observed in Japan (0.41) and South Korea (0.31) [35], which are the only countries that provide a government-sponsored nation-wide screening programme for GC [36]. This difference may suggest that early detection, followed by effective treatment, can significantly improve survival outcomes of GC patients. There is, however, lack of evidence to support the cost-effectiveness of nationwide GC screening programmes in countries where the prevalence of GC is moderate or low. In Taiwan, GC screening is therefore limited to the high-risk population of Matsu island, the offshore island of the country, and recently the population of Changhua County [37]. More effort should be made to develop cost-effective strategies for early detection of GC as well as to provide effective treatments to AGC patients.

While premature death associated with AGC were estimated to be the costliest component of AGC burden in Taiwan, these mortality costs (as well as morbidity costs) were based on the broad definition approach where economic value was placed on a person’s life till the end of his or her life expectancy, not the age of retirement, since the time of death. This approach was supported by recent studies that have criticised the traditional approach – valuing the life years till the age of retirement – as “inaccurate” and “unethical” [9, 26]. Nevertheless, the traditional approach was also employed in our sensitivity analyses. The size of mortality costs, as a share of the total AGC costs, remained substantial, although direct medical costs appeared to greater than mortality costs in the sensitivity analysis. Mortality costs accounted for 40% of the total costs re-estimated, that is US$146 million (about 0.03% of nominal GDP in 2013), and direct medical costs accounted for 47% of the total costs.

Direct medical costs were estimated to be US$26,431 per patient in 2013, incurring about US$69 million at the national-level. Of these, chemotherapy-related costs (chemotherapy drug costs: US$10,912, chemotherapy-related administration/admission costs: US$3941, chemotherapy-related routine examination: US$888) accounted for more than half of the direct medical costs (60%), followed by inpatient costs (29%) and outpatient costs (12%).

Notably, there was no significant difference in direct medical costs as well as direct non-medical costs/morbidity costs between patients who received second-line treatment and those who received BSC following the first-line treatment. Mortality costs could not be estimated by the type of treatment because the costs were estimated based on national mortality statistics (i.e., the actual number of GC-related deaths in Taiwan in 2013). Nevertheless, the previous study [14], using the same chart review data as the one employed here, reported the median survival to be 12.5 months for patients who received second-line treatment and 8.0 months for patients who received BSC, indicating better survival outcomes and supposedly lower mortality costs in patients with second-line treatment. Although this finding should be interpreted with caution because the baseline differences between the two groups were not adjusted for, these real-world findings are consistent with those from recent clinical trials, which have shown better improvement in survival with second-line treatment versus BSC [38–40]. Taken together, it is important to make sure more treatment options are available to patients with AGC; this could reduce the economic burden of AGC in Taiwan through cost savings in mortality costs. Nevertheless, the cost-effectiveness of any new treatment options should be carefully evaluated, given the considerable size of AGC-related direct medical costs.

Finally, the sizes of direct non-medical costs and morbidity costs appeared to be much smaller than those of direct medical costs and mortality costs. Nevertheless, the impact of these costs should not be neglected; these costs are directly related to the welfare of patients and their caregivers because these are the costs fully borne by them. In addition, these costs would be even greater if intangible costs such as psychological distress and subjective burden experienced by patients and caregivers are fully taken into account.

### Limitations

Although this is the first COI study that has estimated the size of AGC burden in Taiwan and can help better prioritise and allocate resources for cancer management, the study suffers from a number of limitations that should be taken into account when interpreting the results. Firstly, this study estimated the burden of AGC based mainly on data from a chart review study previously conducted in Taiwan [14]. While the chart review study provided a useful glimpse of resource utilisation among patients with AGC in actual clinical practice in Taiwan, its findings may not be representative of the treatment for all AGC patients in Taiwan, especially given its small sample size, the quality of chart documentation and study inclusion criteria. The study included only 122 patients, and of these, only 82 patients were included in the final costing analyses due to missing data. Moreover, the accuracy of collected data depends on the quality of chart documentation. While the quality of documentation has improved substantially over the recent years, there is still the possibility of under-documentation on the use of healthcare services that have been made outside of the participating hospitals. Furthermore, the study focused on a specific subset of patients, who had received platinum and/or fluoropyrimidine as first-line therapy, the most widely recommended first-line therapy regimen in Europe and East Asia [41], and had initiated either second-line therapy or BSC. Secondly, the definition of AGC was not fully consistent across the datasets used in this COI study and not always clearly distinguished from that of GC. For instance, given that there was no single definition of AGC uniformly used in the literature [13], we used the same AGC definition as the one employed in the chart review study, which was the main data source for this COI analysis. However, the prevalence data were not based on this definition but based on stage III and IV GC because of data availability. In addition, all GC-related deaths were assumed to be AGC-related deaths because GC patients are likely to experience advanced-stage GC prior to cancer death. This approach would provide equal estimates for both AGC and GC mortality costs. Thirdly, given data constraints, morbidity costs were estimated based only on productivity loss due to spending time on cancer treatment, but not those due to being sick (i.e., absenteeism and presenteeism) and job loss. Nevertheless, the use of a broad definition (i.e., valuing those with unpaid employment, economically inactive, and unemployed) can partially compensate and replace these costs. Finally, doses of chemotherapy drugs were rarely available in the chart review study, and therefore recommended doses, not actual doses, were used to calculate the drug acquisition costs.

## Conclusion

This study confirms that AGC exerts a significant economic burden in Taiwan. The total economic burden of AGC was estimated to be about US$423 million in 2013. This represents 0.08% of the Taiwanese economy (GDP). Mortality costs appeared to be the single greatest contributor to the burden, followed by direct medical costs, morbidity costs and direct non-medical costs. Early detection and providing effective treatments may help to reduce its burden on patients, caregivers and society as a whole.

## Abbreviations

AGC: Advanced Gastric Cancer
BSC: Best Supportive Care
COI: Cost of Illness
DALYs: Disability-Adjusted Life Years
GEJ: Gastroesophageal Junction
NT: New Taiwan
PPP: Purchasing Power Parity
RU: Resource Utilisation
SD: Standard Deviation
US: United States
